# STMN1 as a novel prognostic biomarker in HCC correlating with immune infiltrates and methylation

**DOI:** 10.1186/s12957-022-02768-y

**Published:** 2022-09-20

**Authors:** En-di Zhang, Chenxuan Li, Yuan Fang, Na Li, Zhongyun Xiao, Chuhong Chen, Benkai Wei, Hangping Wang, Jincheng Xie, Yinglei Miao, Zhong Zeng, Hanfei Huang

**Affiliations:** 1 The First Affiliated Hospital of Kunming Medical University, Kunming, 650032 China; 2 Yunnan Province Clinical Research Center for Digestive Diseases, Kunming, 650032 China

**Keywords:** STMN1, Hepatocellular carcinoma (HCC), Diagnosis, Immune infiltration, DNA methylation

## Abstract

**Background:**

Upregulation of Stathmin 1 (STMN1), a cytoplasmic phosphoprotein that controls the dynamics of cellular microtubules, is linked to malignant behavior and poor prognosis in a range of malignancies. However, little research has been done on STMN1’s potential role in HCC as a single factor in DNA methylation, m^6^A, or immunological modulation.

**Results:**

STMN1 is overexpressed in hepatocellular carcinoma, where it is related to clinicopathological parameters and affects the prognosis of HCC patients. STMN1 overexpression plays an important role in the diagnosis and prognosis of hepatocellular carcinoma. Meanwhile, methylation of 7 CpG sites of STMN1 in HCC was correlated with prognosis, and STMN1 expression was closely related to m^6^A modification. In addition, STMN1 expression is associated with immune cell infiltration, immune molecules, and immune checkpoints in HCC.

**Conclusion:**

STMN1 has a significant role in hepatocellular carcinoma diagnosis and prediction. STMN1 is implicated not just in the onset and course but also in the immunological modulation of the disease. DNA methylation and m^6^A are both linked to STMN1. Therefore, STMN1 could be used as a diagnostic and prognostic biomarker for HCC, as well as a target for immunotherapy.

## Background

The most common type of primary liver cancer is hepatocellular carcinoma (HCC) [1]. Every year, about 854,000 new instances of liver cancer are identified, with hepatocellular carcinoma accounting for 85–90% of these cases, making it the world’s sixth most prevalent disease [2]. The majority of people with liver cancer are detected when it has progressed to a late stage. Due to the aggressive nature of hepatocellular carcinoma and its end-stage symptoms, most patients die within 1 year of diagnosis [1, 3]. Surgery, radiation, chemotherapy, immunotherapy, and targeted therapy are now the most common therapies for liver cancer [4]. Although these treatment methods have achieved some clinical success, the prognosis and survival rate of patients with liver cancer are still very poor due to problems such as tumor drug resistance and drug side effects [5]. However, few biomarkers can accurately diagnose liver cancer in the early stage. As a result, finding new therapeutic targets and sensitive tumor biomarkers to identify and treat liver cancer is critical [6].

STMN1 is an oncogene that encodes a highly conserved cytoplasmic phosphorylated protein of 18 kDa [7]. STMN1 protein plays a key role in regulating microtubule dynamics. STMN1 has a tubulin-binding domain, which can sequester α/β Tubulin heterodimers and promote the instability of microtubules [8]. STMN1 promotes cell differentiation, proliferation, and migration, and it is increased in numerous malignancies, including non-small cell lung cancer, breast cancer, and gastric cancer [9]. STMN1 regulates cell proliferation, migration, drug resistance, cancer stem cell characteristics, and tumor growth in vitro and triggers the complex cross talk between liver cells, and the hepatocyte growth factor (HGF)/MET signaling pathway is triggered in hepatic stellate cells (HSC) and hepatic stellate cells (HSC) [8]. In individuals with liver cancer, STMN1 expression was significantly correlated with E2F1/TFPD1 and KPNA2 expression and was associated with poor prognosis in patients with hepatocellular carcinoma [10]. The transcription of STMN1 in the liver is downregulated by T3, suggesting that the lack of normal THR function will lead to the increased expression of STMN1 and the malignant growth of liver cancer [11]. Upregulation of the E2F1 and STMN1 proteins has been linked to poor outcomes in liver cancer patients [12]. In high-expression groups, STMN1 expression is an independent risk factor for multicenter (MC) recurrence [13]. STMN1 affects the epithelial-mesenchymal transformation (EMT) of HCC cells by regulating the dynamic equilibrium of microtubules via the “STMN1 microtubule EMT” axis signal, suggesting that STMN1 might be a viable therapeutic target for limiting liver cancer metastasis [14]. Overexpression of STMN1 has been linked to a poor prognosis in several of the studies mentioned above. However, no research has been done on STMN1’s possible function in DNA methylation, m^6^A, immune cell infiltration, immunological molecules, or immune checkpoints as a single factor.

We investigate the significance of STMN1 in diagnosing and predicting the prognosis of liver cancer, as well as its association with immune cell infiltration, immune cell biomarkers, immunological chemicals, and immune checkpoints, using data from a public scientific database. We examined DNA methylation, m^6^A, and the development and progression of liver cancer.

## Results

### STMN1 expression is higher in cancer than in non-cancer tissues

**Fig. 1:**
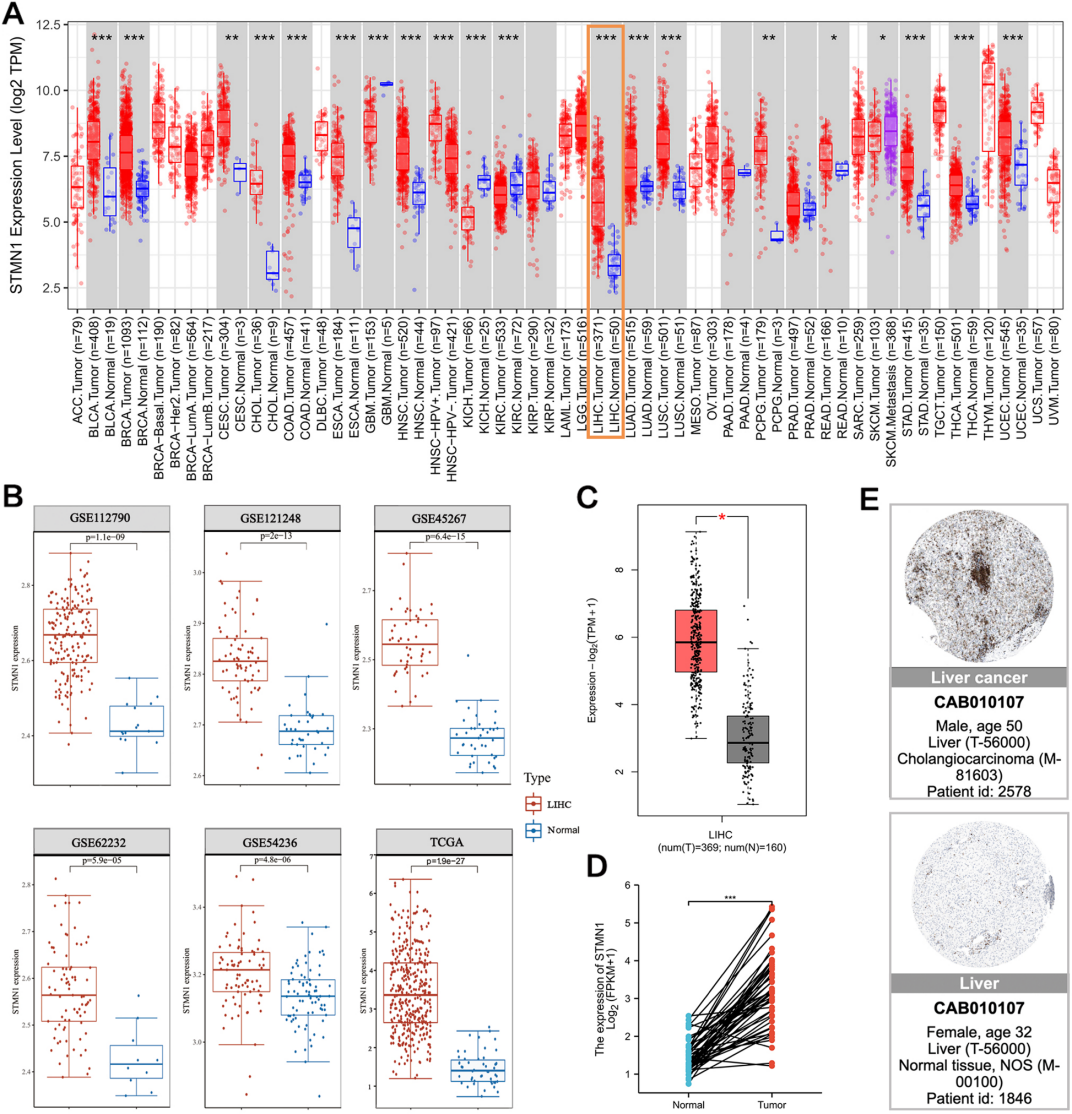
Expression status of STMN1 in cancer. **A** The expression level of STMN1 in 33 cancer tissues and normal tissues (TCGA cancer data compared with TCGA and GTEx normal data). **B** In GSE112790, GSE121248, GSE45267, GSE62232, GSE54236, and TCGA databases, the expression of STMN1 in liver cancer tissues was higher than that in normal tissues. **C** The expression of STMN1 in liver cancer tissues was higher than that in normal tissues in GEPIA2 database (*p* < 0.05). **D** In TCGA database, 50 pairs of HCC tissues and their matched adjacent normal liver tissues, STMN1 expression was higher in HCC tissues (*p* < 0.001). **E** Immunohistochemical staining of clinical liver cancer samples from the HPA database confirmed that STMN1 expression level in tumor tissues was higher than that in normal liver tissues

To investigate STMN1’s potential role in cancer, we first searched for its expression in 33 human cancers (Fig. 1A). STMN1 expression was considerably higher in 19 cancer tissues compared to normal tissues: bladder urothelial carcinoma (BLCA), breast invasive carcinoma (BRCA), cervical squamous cell carcinoma and endocervical adenocarcinoma (CESC), cholangiocarcinoma (CHOL), colonic adenocarcinoma (COAD), esophageal carcinoma (ESCA), glioblastoma multiforme (GBM), head and neck squamous cell carcinoma (HNSC) (KIRC), lung adenocarcinoma (LUAD), prostate adenocarcinoma (PRAD), lung squamous cell carcinoma (LUSC), hepatocellular carcinoma (HCC), stomach adenocarcinoma (STAD), thyroid carcinoma (THCA), and uterine corpus endometrial carcinoma (UCEC).

Following that, we discovered that STMN1 was statistically significantly over-expressed in liver cancer tissues relative to noncancer tissues in six HCC datasets from the GEO and TCGA databases (Fig. 1B). To get reliable results, we validated using the GEPIA2 database and got the same results (Fig. 1C). This result remained consistent in the TCGA database of 50 pairs of HCC samples and matched noncancerous tissues (Fig. 1D). Meanwhile, differences in STMN1 expression between HCC and normal tissues were mirrored in protein expression levels (Fig. 1E). These findings imply that STMN1 may play a role in HCC.

### STMN1 expression is associated with clinicopathological parameters and poor prognosis in HCC patients

**Fig. 2:**
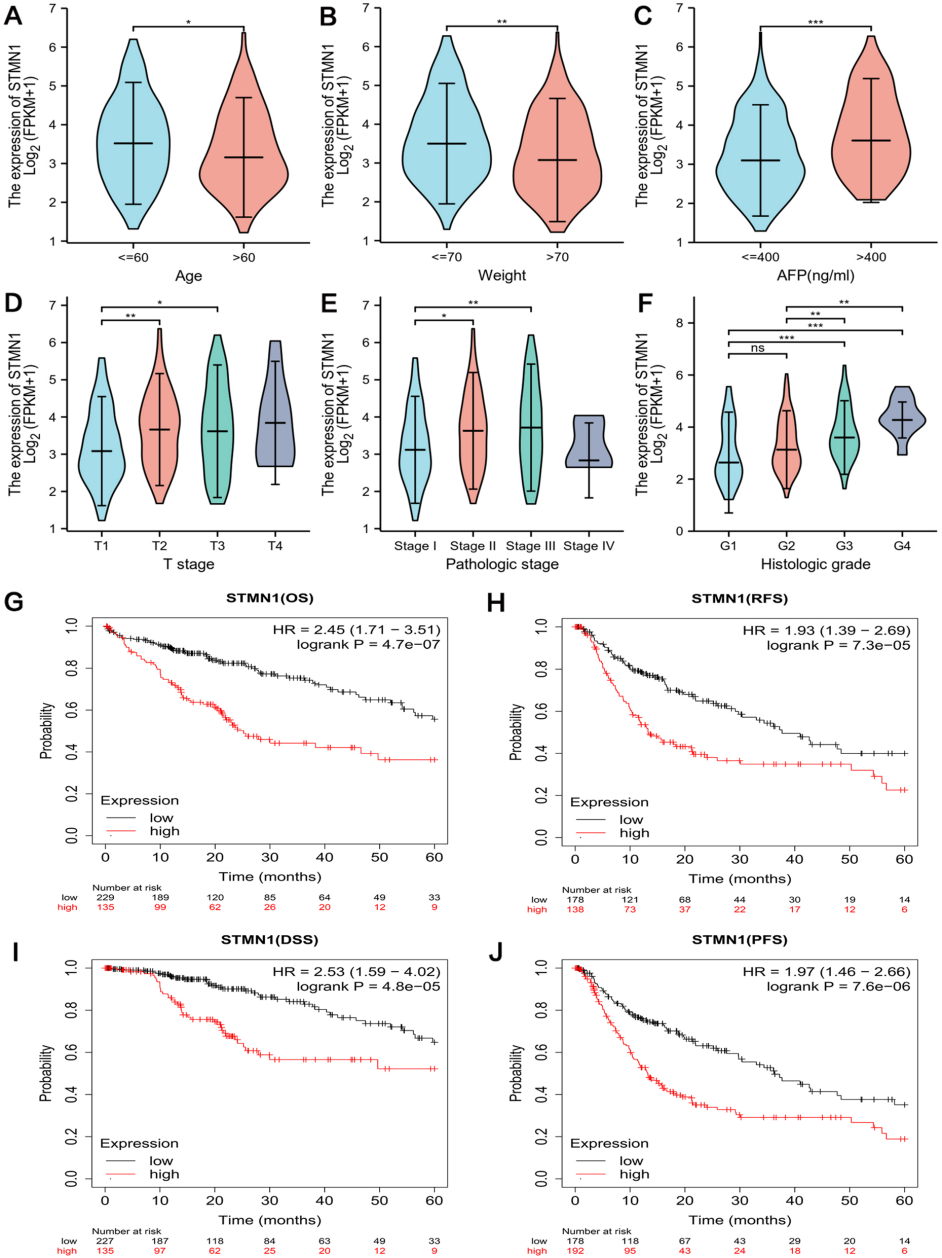
STMN1 expression and clinicopathological parameters and prognosis in HCC patients. The expression level of STMN1 was significantly correlated with age (**A**), body weight (**B**), AFP level (**C**), tumor status (**D**), pathological stage (**E**), and histological grade (**F**). The survival curves of OS (**G**), RFS (**H**), DSS (**I**), and PFS (**J**) of patients with high and low STMN1 expression in HCC were plotted at *p* < 0.05. *p < 0.05, **p < 0.01,***p < 0.001

Because STMN1 is highly expressed in liver cancer, we utilized the TCGA database to investigate the link between STMN1 expression and HCC clinicopathological features. We found that high expression of STMN1 was related to the age, weight, AFP level, tumor status, pathological stage, and histological grade of HCC patients (Fig. 2A–F).

Then, using an Internet database, we looked at STMN1’s prognosis in HCC. High expression of STMN1 was linked with poor overall survival (OS, *p* < 0.001), disease-free survival (RFS, *p* < 0.001), disease-specific survival (DSS, *p* < 0.001), and progression-free survival (PFS, *p* < 0.001) (Fig. 2G–J), according to the Kaplan-Meier survival curve.

High STMN1 expression was shown to be related with a poorer overall survival rate (*HR* = 1.785, 95% *CI* = 1.256–2.536, *p* = 0.001) in a univariate Cox analysis. STMN1 gene expression was found to be an independent risk factor for overall survival in HCC patients (*HR* = 2.009, 95% *CI* = 1.164–3.466, *p* = 0.012) in a multivariate Cox analysis (Table 1).

**Table 1:** Clinical features associated with overall survival by univariate and multivariate cox regression analysis

Characteristics	Total (N)	Univariate analysis		Multivariate analysis	
		Hazard ratio (95% *CI*)	*p*-value	Hazard ratio (95% *CI*)	*p*-value
Age (< = 60 vs. > 60)	373	1.205 (0.850–1.708)	0.295	1.327 (0.811–2.170)	0.260
Tumor status	354	2.317 (1.590–3.376)	**< 0.001**	1.883 (1.105–3.210)	**0.020**
T stage (T1 & T2 vs. T3 & T4)	370	2.598 (1.826–3.697)	**< 0.001**	2.728 (0.153–48.666)	0.495
N stage (N0 vs. N1)	258	2.029 (0.497–8.281)	0.324	2.721 (0.355–20.857)	0.335
M stage (M0 vs. M1)	272	4.077 (1.281–12.973)	**0.017**	1.874 (0.410–8.572)	0.418
Pathologic stage (stage 1 & stage 2 vs. stage 3 & stage IV)	349	2.504 (1.727–3.631)	**< 0.001**	0.882 (0.049–16.023)	0.933
Histologic grade (G1 & G2 vs. G3 & G4)	368	1.091 (0.761–1.564)	0.636	1.092 (0.669–1.782)	0.726
STMN1 (low vs. high)	373	1.785 (1.256–2.536)	**0.001**	2.009 (1.164–3.466)	**0.012**

### STMN1 overexpression’s significance in HCC diagnosis and prognosis

**Fig. 3:**
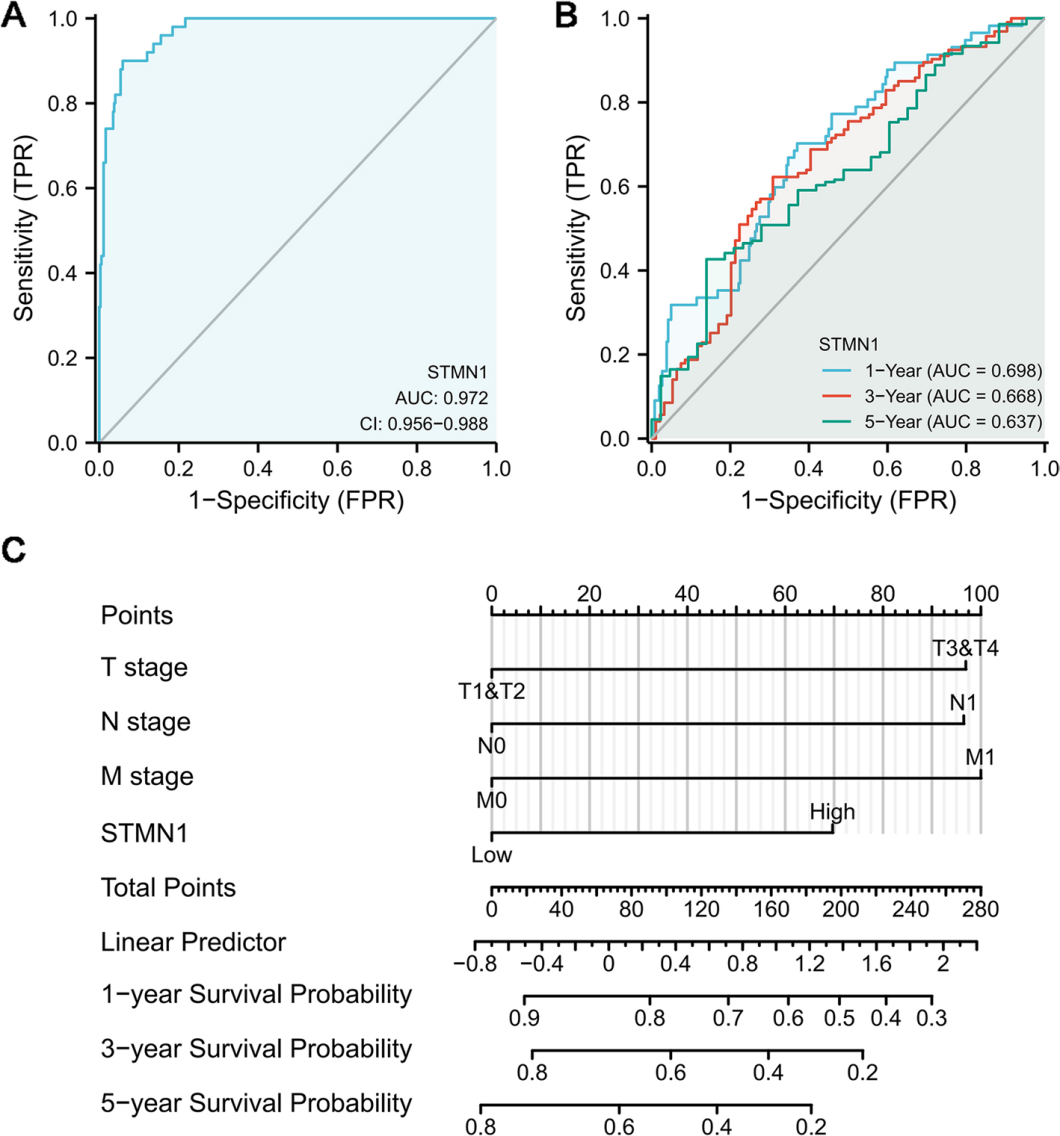
ROC analysis of STMN1 and nomogram model. **A** ROC curve for diagnosis to distinguish tumor from normal tissue. **B** ROC curve analysis of time-dependent survival to predict 1-, 3-, and 5-year survival. **C** A nomogram model combining clinicopathological factors and STMN1 expression levels to predict 1-year, 3-year, and 5-year survival

According to the ROC diagnostic curve, STMN1 expression was able to distinguish cancers from normal tissues with high accuracy (*AUC* = 0.972) (Fig. 3A). To predict 1-, 3-, and 5-year survival, we built a time-dependent ROC survival curve for STMN1. The AUC values for the 1-year, 3-year, and 5-year survival curves were all greater than 0.6, indicating that these data were adequate for prediction (Fig. 3B). We developed a nomogram model to predict the 1-year, 3-year, and 5-year survival probability of patients in clinical practice by combining clinicopathological parameters (including stages T, N, and M) and STMN1 levels (Fig. 3C). STMN1 expression has a high capacity to predict 1-, 3-, and 5-year survival rates, according to the findings.

### Correlation analysis of STMN1 with methylation and m^6^A genes in HCC patients

**Fig. 4:**
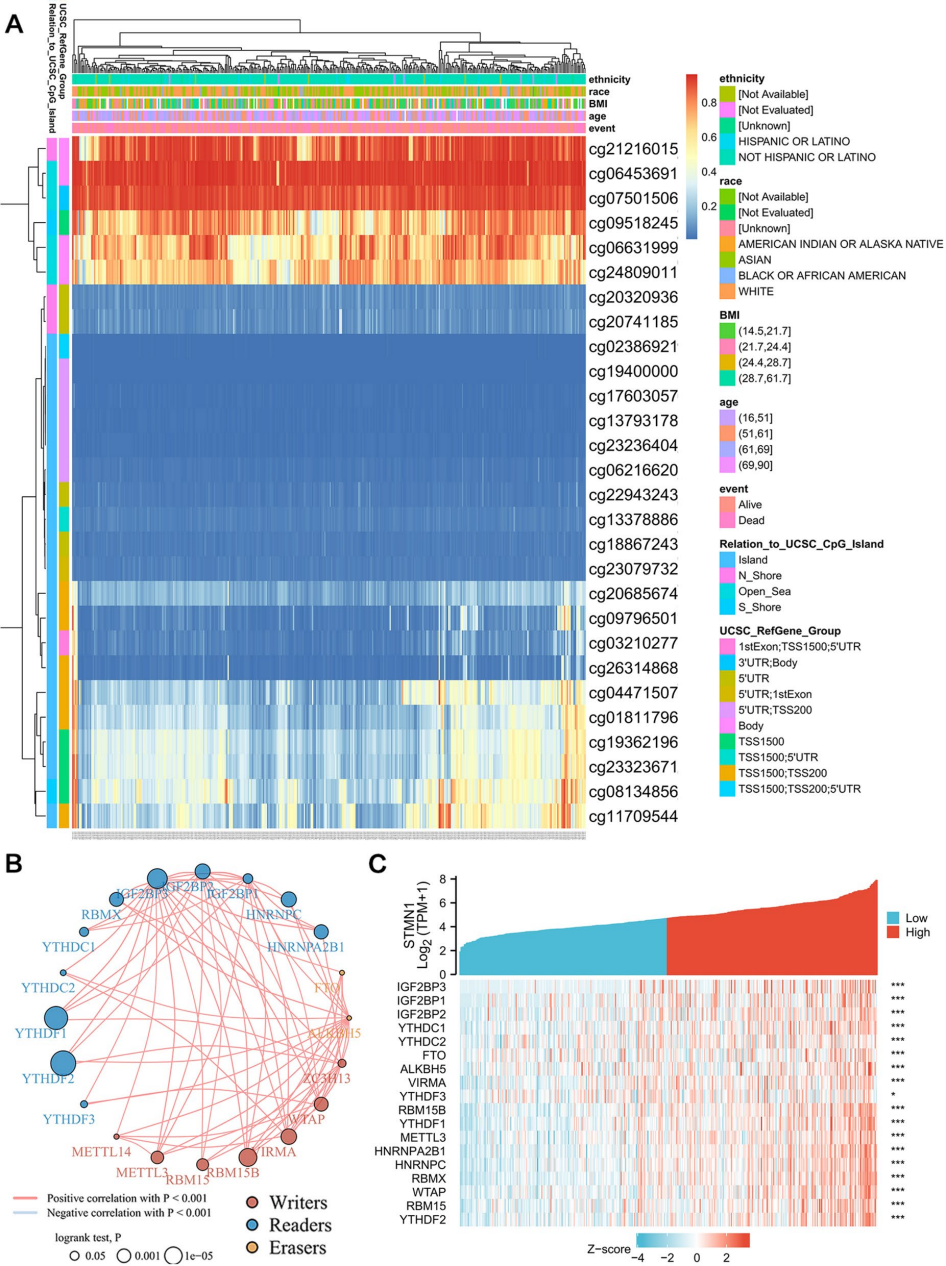
Correlation of STMN1 with methylation and m^6^A genes. **A** Visualization between methylation levels and STMN1 expression. **B** Correlation between m^6^A-related genes. **C** Differential expression of m^6^A-related genes in the high and low STMN1 expression groups of HCC samples

The DNA methylation level of STMN1 with a predictive value of CpG was investigated using the MethSurv tool. MethSurv showed 29 CpG methylation sites, among which CG21216015, CG06453691, and CG07501506 had the highest DNA methylation (Fig. 4A). The methylation levels of seven CpG sites were correlated with prognosis, namely CG07501506, CG09796501, CG13793178, CG21216015, CG23079732, CG24809011, and CG26314868 (*p* < 0.05) (Table 2). Patients with high STMN1 methylation at these CpG loci had a lower survival rate than those with low STMN1 methylation.

**Table 2:** Effect of hypermethylation level on prognosis in HCC

CpG	HR	*p*-value
3′UTR-Open_Sea-cg07501506	1.897	0.0063
TSS1500-Island-cg09796501	0.668	0.0426
TSS200-Island-cg13793178	1.751	0.0016
Body-N_Shore-cg21216015	1.545	0.0140
5′UTR-Island-cg23079732	0.609	0.0057
Body-Open_Sea-cg24809011	0.673	0.0421

M^6^A mutations have a key role in the incidence and progression of HCC. Therefore, we conducted a correlation analysis of STMN1 and m^6^A-related genes. Using the TCGA database, we looked at the relationship between the expression of 20 m6A-related genes in liver cancer, and the results indicated that when compared to normal liver tissue, the expression of 20 m^6^A-related genes was higher in liver cancer, IGF2BP3, IGF2BP1, IGF2BP2, YTHDC1, YTHDC2, FTO, ALKBH5, VIRMA, YTHDF3, RBM15B, YTHDF1, METTL3, HNRNPA2B1, HNRNPC, RBMX, WTAP, RBM15, and YTHDF2; the expression of these m^6^A-related genes was significantly increased (Fig. 4B). Subsequently, we continued to analyze the expression differences of these m^6^A genes increased in liver cancer between the high-expression STMN1 group and the low-expression STMN1 group to determine whether the m^6^A modifications were different between the high-expression STMN1 and the low-expression STMN1 group. We were surprised to find that in the STMN1 high-expression group, IGF2BP3, IGF2BP1, IGF2BP2, YTHDC1, YTHDC2, FTO, ALKBH5, VIRMA, RBM15B, YTHDF1, METTL3, HNRNPA2B1, HNRNPC, RBMX, WTAP, RBM15, and YTHDF2 were significantly expressed upregulated (*p* < 0.001) (Fig. 4C). Therefore, these results suggest that STMN1 is closely related to m^6^A modification in HCC.

### Correlation between STMN1 and immune cell infiltration

**Fig. 5:**
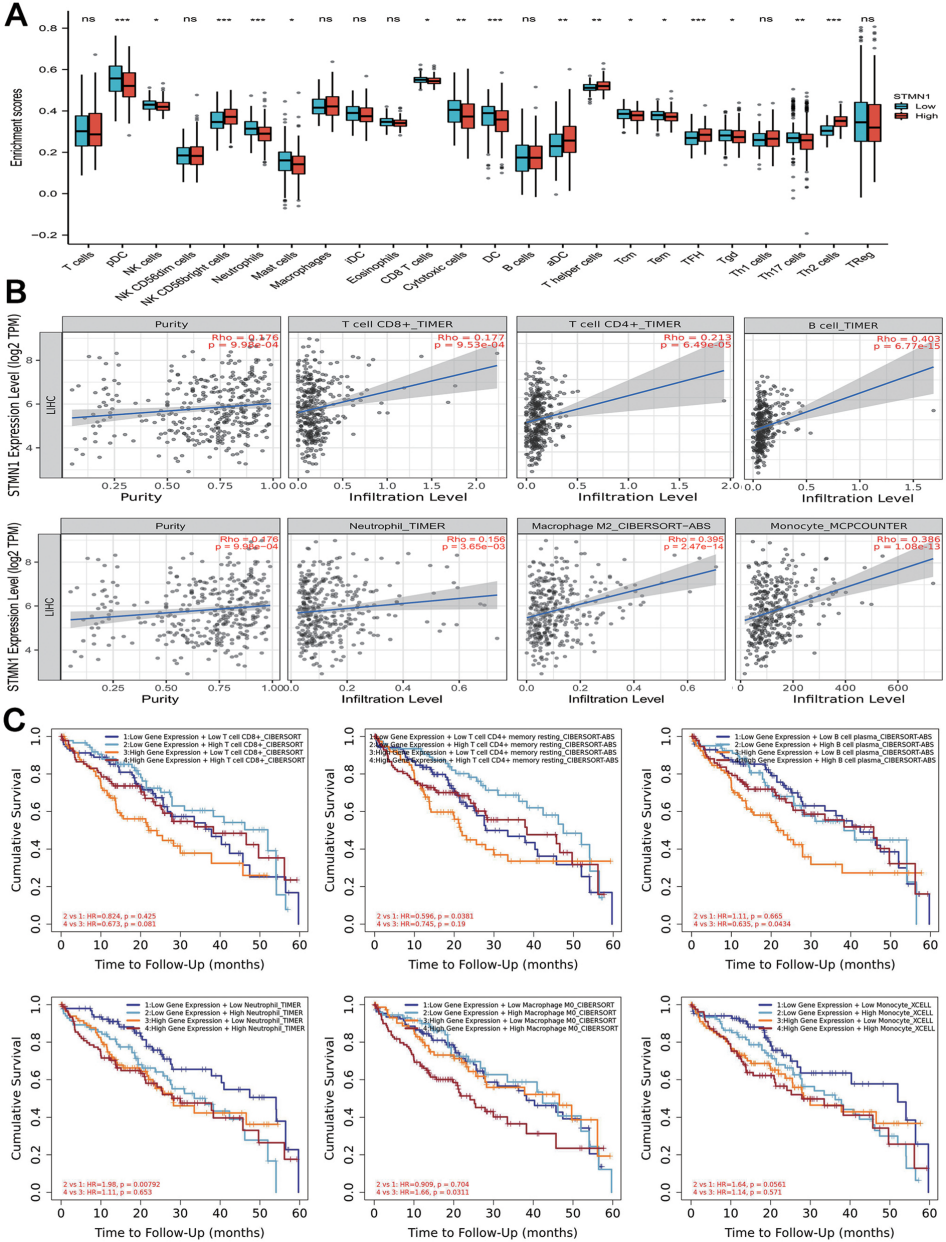
Correlation between STMN1 and immune cell infiltration. **A** Immune infiltration between STMN1 high-expression group and STMN1 low expression group. **B** The expression of STMN1 was significantly correlated with the infiltration of immune cells. **C** Cumulative survival between high and low levels of STMN1 expression under different immune cell infiltrations

We subsequently looked at the immune cell infiltration status in HCC to see if there was a link between STMN1 expression and immune cell infiltration. The immune infiltration of the STMN1 high-expression group and the STMN1 low expression group was compared using 187 HCC samples from the TCGA database. We performed analysis using the ssGSEA technique and found that STMN1 expression was strongly associated with the level of infiltration of pDC, NK CD56 bright cells, neutrophils, DC, TFH, and Th2 cells (Fig. 5A, *p* < 0.01).

STMN1 expression was strongly linked with tumor purity (Rho = 0.176, *p* = 9.98E-04) in the TIMER 2.0 database. In addition, we found that STMN1 expression was linked to immune cell infiltration, particularly T-cell CD8+ T cells (*r* = 0.177, *p* = 9.53E-04), T-cell CD4+ (*r* = 0.213, *p* = 6.49E-05), B cell (*r* =0.403, *p* = 6.77E-15), neutrophil (*r* = 0.156, *p* = 3.65E-03), macrophage (*r* = 0.536, *p* = 4.88E-27), monocyte (*r* = 0.386, *p* = 1.08E-13) (Fig. 5B).

Finally, we examined the cumulative survival rate of immune cells with high and low STMN1 expression levels under various immune cell infiltration settings. CD8+ T cells and B cells with high STMN1 expression had a better prognosis, while CD4+ T cells with low STMN1 expression had a worse prognosis, according to the findings. Low STMN1 expression was associated with a better prognosis in neutrophils and monocytes, whereas high STMN1 expression was associated with a worse prognosis in macrophages (Fig. 5C).

We utilized the TIMER database to investigate the relationship between STMN1 expression and liver cancer immune cell biomarkers in order to learn more about STMN1’s function in the tumor immune microenvironment (Table 3). The results showed that STMN1 was associated with CD8+ B-cell biomarkers (CD19, CD27, CD38, and CD79A) and T-cell biomarkers (CD3D, CD3E, and CD2) in HCC T-cell biomarkers (CD8A, CD8B); other T-cell subsets (Tfh, Th1, Th2, Th9, Th22, and Treg), exhausted T cell, M1 macrophage biomarkers (IRF5, PTGS2), M2 macrophage biomarkers (CD163, VSIG4, MS4A4A), TAM biomarkers (PDCD1LG2, CD80, CD40, TLR7, CCL2, and IL10), monocytes (CD86, CSF1R)), natural killer cell biomarkers (NCAM1, KIR2DL3, KIR2DL4, KIR3DL2, KIR2DS4, CD314, CD7, and XCL1), neutrophil biomarkers (ITGAM, FUT4, and MPO), and dendritic cell biomarkers (CD1C, HLA-DPB1, HLA-DQB1, HLA-DRA, HLA-DPA1, NRP1, and ITGAX) were significantly positively correlated. As a result, these findings show that STMN1 is closely linked with immune cell infiltration.

**Table 3:** Correlation analysis between STMN1expression and immune cell markers in HCC

Immune cells	Biomarkers	None		Purity	
		Cor	*P*	Cor	*P*
B cell	CD19	0.309	***	0.375	***
	CD27	0.262	***	0.390	***
	CD38	0.239	***	0.343	***
	CD79A	0.163	***	0.278	***
T cell (general)	CD3D	0.271	***	0.390	***
	CD3E	0.209	***	0.365	***
	CD2	0.209	***	0.351	***
CD8+ T cell	CD8A	0.238	***	0.351	***
	CD8B	0.231	***	0.336	***
Tfh	CXCR3	0.269	***	0.385	***
	CXCR5	0.191	***	0.300	***
	BCL6	0.131	**	0.119	**
	ICOS	0.283	***	0.400	***
Th1	IFN-γ (IFNG)	0.296	***	0.376	***
	TNF-α (TNF)	0.230	***	0.347	***
	IL12RB2	0.215	***	0.247	***
	STAT4	0.240	***	0.304	***
	STAT1	0.333	***	0.370	***
	CD94 (KLRD1)	0.098	**	0.178	***
	BET (TBX21)	0.123	**	0.224	***
Th2	STAT6	0.150	***	0.129	**
	CCR3	0.262	***	0.329	***
	CD4	0.274	***	0.365	***
	STAT5A	0.291	***	0.337	***
Th9	IRF4	0.221	***	0.337	***
	SPI1	0.337	***	0.499	***
Th22	CCR10	0.446	***	0.479	***
Treg	CD25 (IL2RA)	0.247	***	0.383	***
	CCR8	0.313	***	0.392	***
	FOXP3	0.146	***	0.217	***
Exhausted T cell	PD-1 (PDCD1)	0.301	***	0.403	***
	Tim-3 (HAVCR2)	0.319	***	0.470	***
	CTLA4	0.327	***	0.441	***
	LAG3	0.328	***	0.376	***
	GZMB	0.156	***	0.220	***
M1 macrophage	INOS (NOS2)	−0.019	0.712	−0.012	0.829
	IRF5	0.354	***	0.338	***
	COX2 (PTGS2)	0.080	0.125	0.202	***
M2 macrophage	CD163	0.111	***	0.216	***
	ARG1	−0.164	***	−0.162	***
	VSIG4	0.131	**	0.239	***
	MS4A4A	0.146	***	0.278	***
TAM	CD80	0.336	***	0.443	***
	PDCD1LG2	0.128	**	0.241	***
	CD40	0.299	***	0.312	***
	TLR7	0.248	***	0.362	***
	CCL2	0.097	**	0.208	***
	IL10	0.201	***	0.302	***
Monocyte	CD86	0.316	***	0.468	***
	CD115 (CSF1R)	0.194	***	0.334	***
NK cell	NCAM1	0.234	***	0.315	***
	KIR2DL1	0.008	0.871	−0.025	0.649
	KIR2DL3	0.209	***	0.255	***
	KIR2DL4	0.268	***	0.294	***
	KIR3DL1	0.070	0.176	0.076	0.158
	KIR3DL2	0.135	***	0.180	***
	KIR2DS4	0.116	**	0.105	**
	CD314 (KLRK1)	0.153	***	0.263	***
	CD7	0.279	***	0.369	***
	XCL1	0.392	***	0.433	***
Neutrophil	CD11b (ITGAM)	0.287	***	0.370	***
	CD15 (FUT4)	0.338	***	0.372	***
	MPO	0.112	**	0.160	***
Dendritic cell	CD1C	0.100	**	0.198	***
	HLA-DPB1	0.220	***	0.337	***
	HLA-DQB1	0.188	***	0.294	***

*p < 0.05; **p < 0.01; ***p < 0.001

### Correlation between STMN1 and immune molecules

**Fig. 6:**
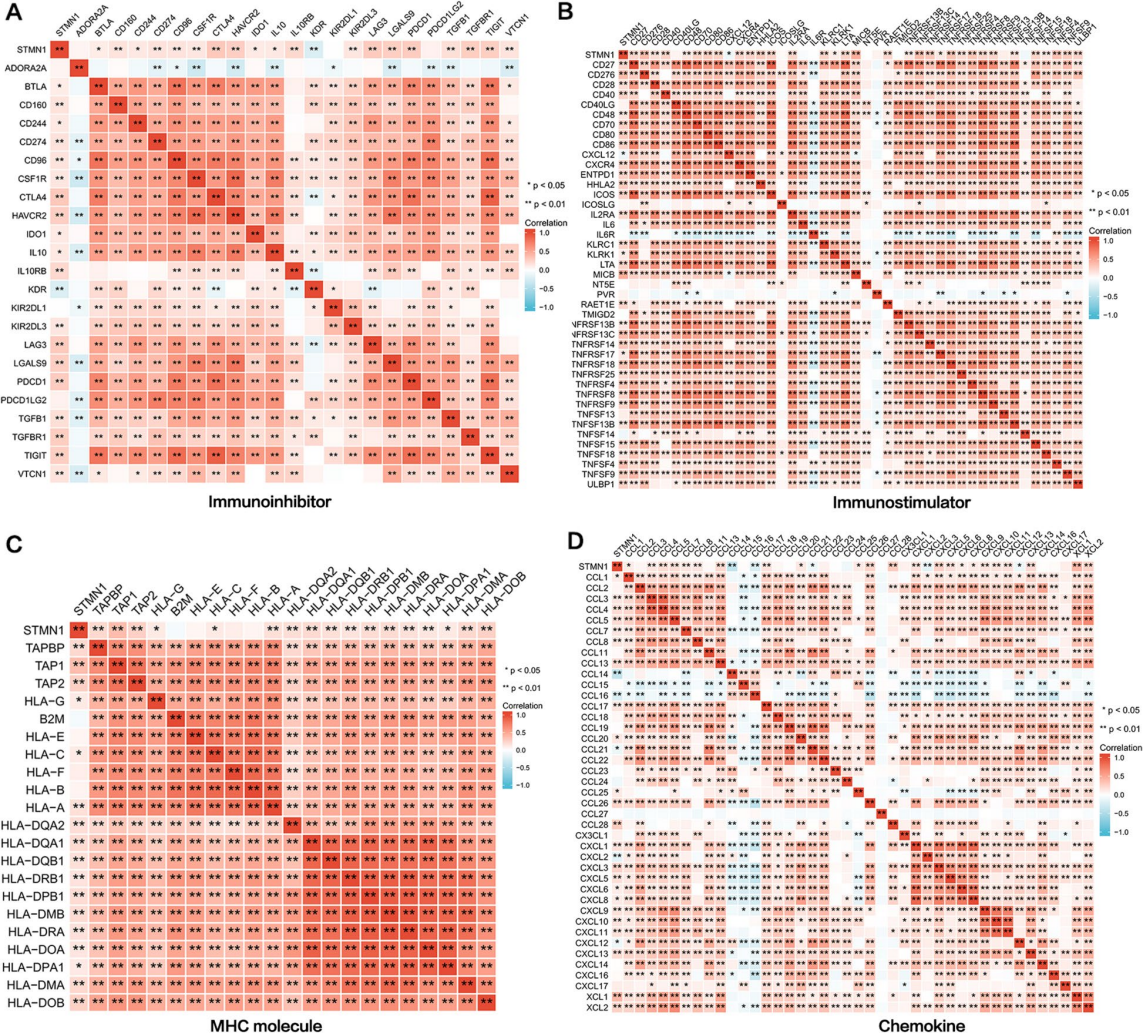
Correlation between STMN1 expression level and HCC immune modulator, MHC molecule, and chemokine. **A**–**B** Correlation between STMN1 expression level and immunostimulants and immune inhibitors. **C** Correlation between STMN1 expression level and MHC molecules. **D** Correlation between STMN1 expression level and chemokines

We next examined the association between STMN1 expression and a variety of immunological-related markers, such as immune modulators and chemokines, to learn more about the link between STMN1 and immune infiltration. The TCGA database was used to determine the connection between STMN1 expression and other immune-related markers. Immunomodulators are further divided into immunostimulants, immune inhibitors, and major histocompatibility complex (MHC) molecules. STMN1 expression was shown to associate well with most immunomodulators and chemokines (Fig. 6A–D).

Therefore, it was confirmed that STMN1 is extensively involved in the regulation of various immune molecules in HCC to influence immune invasion in the tumor microenvironment.

### The relationship between STMN1 expression and immune checkpoint in HCC

**Fig. 7:**
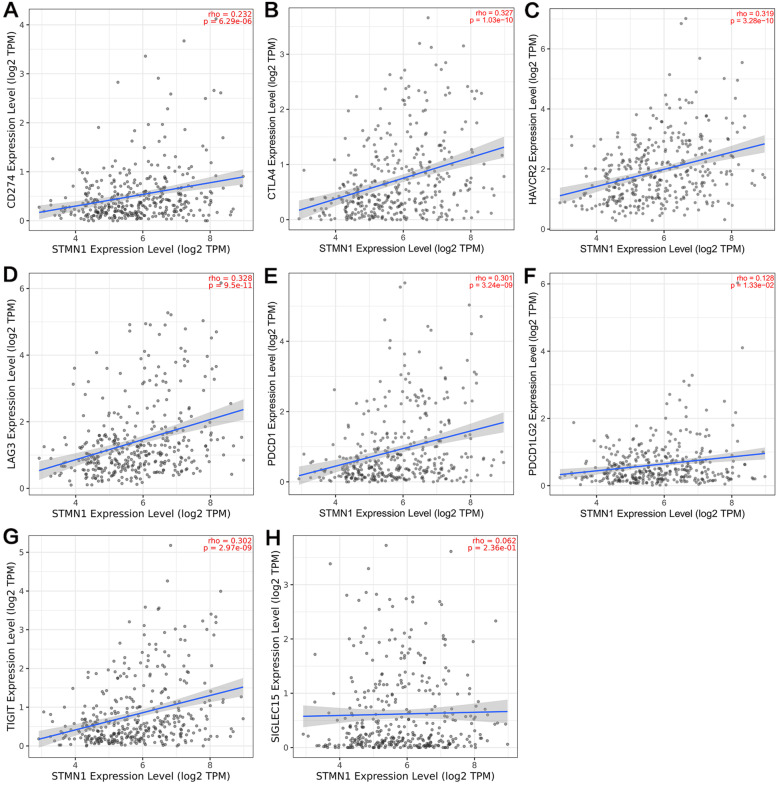
Correlation between STMN1 expression and immune checkpoint-related gene expression in HCC

CD274, CTLA4, HAVCR2, LAG3, PDCD1, PDCD1LG2, TIGIT, and SIGLEC15 are genes associated with the immune checkpoint. Considering the potential carcinogenic role of STMN1 in liver cancer, STMN1 and these 8 immune checkpoint-related genes in HCC were investigated using the TIMER database. We found that STMN1, CD274, CTLA4, HAVCR2, LAG3, PDCD1, PDCD1LG2, and TIGIT were significantly positively correlated in HCC (Fig. 7A–H).

### Identification and validation of PPI network and Hub genes

**Fig. 8:**
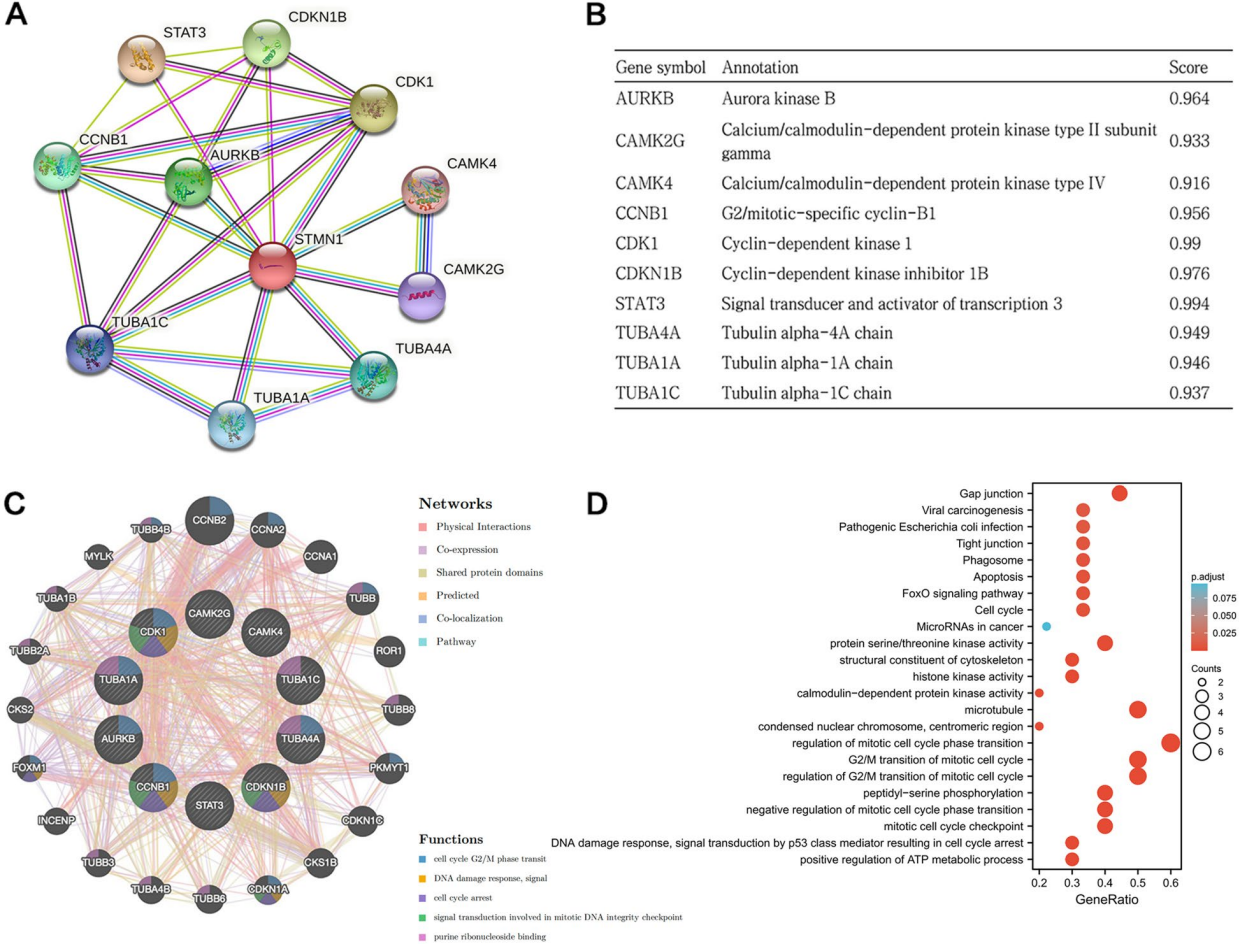
Protein-protein interaction (PPI) networks of hub genes. **A** PPI network of the first 10 hub genes created by STRING. **B** Annotation of 10 functional partner genes of STMN1. **C** PPI networks and function analyses of the 11 upregulated hub genes. Inner circles represent the input genes, and outer circles correspond to gene mania proposed by hub genes; the size of the circles indicates the correlation with the input genes. **D** GO and KEGG enrichment analyses of STMN1 and functional partner genes in HCC

Based on the STRING database, the top ten most relevant functional partner genes were selected to construct the PPI network of STMN1 (Fig. 8A). These genes are AURKB, CAMK2G, CAMK4, CCNB1, CDK1, CDKN1B, STAT3, TUBA1A, TUBA1C, and TUBA4A (Fig. 8B). We constructed a gene-gene interaction network for these genes using the GeneMANIA database to analyze the function of these genes. The central node representing the above genes is surrounded by 20 gene nodes significantly associated with the above genes (Fig. 8C). GO and KEGG were used to investigate these genes. The results of a GO enrichment study revealed that there were significant differences in “regulation of G2/M transition of the mitotic cell cycle,” “regulation of mitotic cell cycle phase transition,” “G2/M transition of mitotic cell cycle,” “microtubule,” “condensed nuclear chromosome, centromeric region,” “histone kinase activity,” “structural constituent of cytoskeleton,” and “protein serine/threonine kinase activity.” According to KEGG pathway analysis, these genes are mostly engaged in “gap junction,” “cell cycle,” “fox-O signaling pathway,” “apoptosis,” and “phagosome” (Fig. 8D, Table 4).

**Table 4:** GO and KEGG enrichment analyses of STMN1 and functional partner genes in HCC

Ontology	ID	Description	*p*-value
BP	GO:0010389	Regulation of G2/M transition of mitotic cell cycle	2.93e-08
BP	GO:1901990	Regulation of mitotic cell cycle phase transition	3.39e-08
BP	GO:0000086	G2/M transition of mitotic cell cycle	9.29e-08
BP	GO:0007093	Mitotic cell cycle checkpoint	1.19e-06
BP	GO:1903580	Positive regulation of ATP metabolic process	1.77e-06
BP	GO:0006977	DNA damage response, signal transduction by p53 class mediator resulting in cell cycle arrest	3.02e-06
BP	GO:1901991	Negative regulation of mitotic cell cycle phase transition	5.99e-06
BP	GO:0018105	Peptidyl-serine phosphorylation	1.25e-05
CC	GO:0005874	Microtubule	9.43e-07
CC	GO:0000780	Condensed nuclear chromosome, centromeric region	7.48e-05
MF	GO:0035173	Histone kinase activity	8.80e-08
MF	GO:0005200	Structural constituent of cytoskeleton	2.17e-05
MF	GO:0004674	Protein serine/threonine kinase activity	6.97e-05
MF	GO:0004683	Calmodulin-dependent protein kinase activity	1.08e-04
KEGG	hsa04540	Gap junction	1.59e-06
KEGG	hsa04110	Cell cycle	2.77e-04
KEGG	hsa04068	FoxO signaling pathway	3.26e-04
KEGG	hsa04210	Apoptosis	3.64e-04
KEGG	hsa04145	Phagosome	5.05e-04
KEGG	hsa04530	Tight junction	6.89e-04
KEGG	hsa05130	Pathogenic *Escherichia coli* infection	0.001
KEGG	hsa05203	Viral carcinogenesis	0.001
KEGG	hsa05206	MicroRNAs in cancer	0.044

## Discussion

STMN1 affects cell cycle progression and microtubule dynamics [15]. It is an oncogene that is highly expressed in a variety of human tumors and has been linked to malignant behavior and a bad prognosis in a number of them. STMN1 overexpression in liver cancer has previously been linked to invasion on the local level, early recurrence, and a bad prognosis, as well as facilitating the polyploid formation and other biological functions [9]. However, the studies related to STMN1 in HCC are currently inadequate. In the present study, we explored the relationship between STMN1 in liver cancer function enrichment, immune infiltration, DNA methylation, and m^6^A, providing new evidence that STMN1 is a prognostic marker that can be exploited as a therapy target for HCC.

In this research, we discovered that HCC had considerably higher STMN1 expression than normal tissues, and that this difference was mirrored at the protein level. Furthermore, increased STMN1 expression was linked to HCC patients’ age, gender, AFP level, tumor status, clinical stage, and histological grade. These findings suggest that STMN1 is involved in the initiation and/or development of HCC. Meanwhile, survival analysis revealed that elevated STMN1 expression was linked to a poor prognosis in HCC patients, with poor OS, RFS, DSS, and PFS. Nomogram model results demonstrate that STMN1 expression can predict 1-, 3-, and 5-year survival rates, suggesting that STMN1 has the potential to be a useful diagnostic and prognostic biomarker in liver cancer. STMN1 expression is now connected to a poor prognosis in a number of cancers [16–18], as well as cancer curability, recurrence, and resistance to adjunctive chemotherapy. STMN1 stimulates the formation, growth, and proliferation of HCC cells through upregulation by FoxM1, and the combination of STMN1 and FoxM1 could become a more accurate predictive biomarker [19]. Current studies suggest that upregulation of STMN1 can accelerate the formation and/or progression of hepatocellular carcinoma by activating the YAP1 signaling pathway, and overexpression of STMN1 may be a precursor of hepatocellular carcinoma and can be used as a marker for diagnosis and treatment [20].

Aberrant DNA methylation is an epigenetic mechanism that can be observed in all types of cancer. We looked at the link between STMN1 DNA methylation levels and HCC patient prognosis. Methylation levels at seven CpG sites, including CG07501506, CG13793178, and CG21216015, which had the greatest levels of DNA methylation, were linked to a worse prognosis. M6A regulates RNA transcription, splicing, processing, translation, and degradation through RNA methylation. It plays a role in the occurrence and spread of a range of malignant tumors, acting as an oncogene or anticancer gene [21]. As a result, the connection between STMN1 expression and RNA methylation in HCC was also studied. We discovered that in the STMN1 high-expression group, RNA methylation-related gene expression was dramatically increased, indicating that STMN1 expression in HCC is linked to m6A alteration. Studies have shown that DNA methylation of STMN1 has a potential relationship with cancer recurrence and prognosis [22]. There is mounting evidence that methylation plays a critical role in cancer through a variety of processes, opening up new avenues for cancer detection and therapy. The immune system plays a crucial role in the origin and progression of malignant tumors [23]. Understanding how tumors and immune cells interact will aid in the prediction of immunotherapy responses and the development of new immunotherapy targets [24]. HCC is an inflammatory malignancy in which the immune system has a role in its development, progression, metastasis, and recurrence [25]. As a result, we looked at how STMN1 correlated with immune cell infiltration, immunological molecules, and immune checkpoints. STMN1 expression was shown to be largely associated with immune cell infiltration, including CD8+ T cells, CD4+ T cells, B cells, neutrophils, macrophages, and monocytes, in our research. Simultaneously, we discovered that STMN1 expression was associated with immune modulators and chemokines, indicating that STMN1 is engaged in regulating a variety of immune components in HCC to influence immunological invasion of the tumor microenvironment. Immune checkpoint molecules are immune system inhibitory regulatory molecules that are necessary for maintaining tolerance, avoiding autoimmune reactions, and modulating the timing and severity of immune responses [26]. Cancer cells manipulate immunological checkpoint molecules to elude immune monitoring during immune editing [27]. Previous research has demonstrated that checkpoint blocking cancer immunotherapy works by inhibiting tumor cells with antibodies produced by the checkpoint inhibitor PD-L1, activating the immune system, and infiltrating the tumor with immunoactive T lymphocytes [28]. The immunological checkpoint molecules CD274, CTLA4, HAVCR2, LAG3, PDCD1, PDCD1LG2, and TIGIT were discovered to have a substantial positive correlation with STMN1 expression. Immunotherapy targeting immunological checkpoints such CTLA4, PDCD1 (PD-1), and PDCD1LG2 (PD-L2) has revolutionized the treatment of many solid cancers, according to previous research [29]. TIL burden is related with upregulation of important immunological checkpoint genes (e.g., CTLA4, PDCD1, PDCD1LG2, CD274), which suppresses T-cell activation [30]. These results suggest that STMN1 affects immune invasion in the tumor microenvironment and may provide a new direction and target for immunotherapy of liver cancer.

STMN1 and Hub genes were shown to be involved in mitotic cell cycle regulation, microtubules, gap junction, FoxO signaling pathway, and apoptosis, according to GO and KEGG enrichment analysis. The mitotic index is an additional prognostic parameter that could provide additional information for patients’ outcomes [31]. Knocking down STMN1 in cancer cells leads to cell cycle stagnation in the G2/M phase, thereby increasing tumor sensitivity to paclitaxel and vincristine [32]. Vincristine is now utilized in the treatment of liver cancer in clinical trials. As a result, it needs to be seen if the STMN1 gene is linked to chemotherapeutic medications like vincristine in the treatment of liver cancer. STMN1 is controlled by KPNA2 via E2F1/TFDP1, establishing a new functional and prognostic relationship between HCC nuclear transport and microtubule (MT) interaction proteins [10]. The current study showed that STMN1 is a prognostic predictor of esophageal squamous cell carcinoma and a marker of PI3K pathway activation [33]. Furthermore, STMN1 overexpression is associated with upregulation of FOXM1 in patients with advanced non-small cell lung cancer, and STMN1/FOXM1 upregulation leads to poor prognosis [34]. Also, STMN1 expression was an independent prognostic factor in patients with early-stage lung adenocarcinoma but only in patients with early-stage cancer [35]. These findings confirm that STMN1 has a role in the progression of human cancers. Meanwhile, these findings also expand new thinking and direction for the mechanism studies of STMN1 in hepatocellular carcinoma.

Although we performed a comprehensive and systematic analysis of STMN1 and validated it using different databases, our study still has some limitations. First, the microarray and sequencing data from different databases have differences and lack specificity, leading to systematic errors. Second, we need to perform ex vivo experiments to validate the potential biological functions of STMN1, which will improve the credibility of our results, which is our next upcoming step. Third, although our results suggest that STMN1 is closely associated with immune cell infiltration in hepatocellular carcinoma, we do not have direct evidence that STMN1 is directly involved in immune regulation. Finally, we do not have complete cases and data to show the effectiveness of targeted drugs against STMN1 in liver cancer treatment, but in the future, we will conduct further series of experiments to improve the shortcomings of our above studies and develop novel immunotherapeutic drugs for the manifestation of STMN1 in liver cancer.

## Conclusion

STMN1 expression was greater in HCC tissues than in normal tissues, and it was linked to clinicopathological variables. Upregulation of STMN1 expression is related with a poor prognosis in patients, suggesting that it might be a useful biomarker for HCC diagnosis and prognosis. The methylation of STMN1 is linked to the prognosis of HCC, and STMN1 expression is tightly linked to the alteration of m6A. STMN1 was found to be linked with B cells, CD8+ T cells, CD4+ T cells, macrophages, neutrophils, and monocytes in tumor immune infiltration. STMN1 expression levels are linked to immune modulators and chemokines, and it is implicated in the regulation of a variety of immunological components in HCC. STMN1 expression is favorably linked with important immune checkpoint molecules, suggesting that it might be a potential target for liver cancer immunotherapy. Because there are few studies on STMN1’s immune microenvironment in HCC, more research on STMN1 in HCC is needed to progressively explain the biological activities of STMN1 in the immune microenvironment and HCC patients’ prognosis.

## Methods

### TCGA database

The Cancer Genome Atlas (TCGA) (https://genome-cancer.ucsc.edu/) is a large, free reference database for cancer research. From the TCGA database, we collected RNA seq data in level 3 HTS eq-FPKM format as well as clinical data on HCC patients. TCGA provided 374 HCC samples with prognostic information and 50 normal liver tissue samples.

### GEO database

The GEO database (https://www.ncbi.nlm.nih.gov/gds) is a free storage database for second-generation sequencing and other high-throughput sequencing data. It offers tools to enable users query and download the gene expression profiles. We downloaded five data sets (GSE112790, GSE121248, GSE45267, GSE62232, and GSE54236) from the GEO database for gene expression analysis.

### GEPIA2

Gene expression profile interaction analysis (GEPIA2) is a database for gene expression and interaction analysis in cancer and normal tissues (http://gepia2.cancer-pku.cn/#index). There were 369 HCC tissue samples and 160 normal tissue samples in the GEPIA database. The gene expression differential analysis was validated using the GEPIA database.

### HPA (Human Protein Atlas) database

The HPA (Human Protein Atlas) (https://www.proteinatlas.org/) is based on immunohistochemistry data in the database’s proteomics, transcriptome, and system biology data. The HPA database was used to investigate the amount of STMN1 protein expression in liver tissue.

### Database of Kaplan-Meier plotters

The Kaplan and Meier plotter (http://kmplot.com/analysis/) is a free, user-friendly online survival analysis tool that includes 54,675 genes and 18,674 cancer samples. In HCC patients, the Kaplan-Meier plotter database was utilized to assess the connection between clinical survival prognosis and STMN1 expression.

### MethSurv database

The MethSurv database (https://biit.cs.ut.ee/methsurv/) is a network based on CpG methylation patterns for survival analysis tools, including 25 distinct forms of methylation in human cancer data utilizing Cox proportional hazards models. The DNA methylation locations of STMN1 in the TCGA database were analyzed using the MethSurv database. CpG methylation in STMN1 was also evaluated for its predictive significance.

### The TIMER database

The TIMER (https://cistrome.shinyapps.io/timer/) is an RNA-seq expression profile data analysis of immune cells in the tumor tissue infiltration database using high-throughput sequencing. B cells, CD4+ T cells, CD8+ T cells, neutrophils, macrophages, and monocytes are the primary infiltrating cells. In many kinds of cancer, TIMER may also be used to investigate gene expression in tumor tissue and normal tissue. The relationship of STMN1 with biomarkers of tumor immune infiltrating cells and liver cancer immune cells was investigated using the TIMER database.

### The STRING database

The STRING database (https://string-db.org/) is an online database that searches for known protein interactions, including both direct physical interactions and indirect functional connections. The STRING database not only generates elegant protein-protein interaction (PPI) diagrams but also provides functional enrichment analysis of common proteins, reference publications, etc. The STRING database was used to investigate STMN1’s protein-protein interaction (PPI) network.

### The GeneMANIA database

The GeneMANIA database (http://genemania.org/search/) is used to generate hypotheses about gene function, analyze gene lists, and prioritize genes for functional analysis. We used the GeneMANIA database to construct gene-gene interaction networks for genes associated with STMN1 function to assess the functions of these genes.

### GO and KEGG database

Gene Ontology (GO) (http://geneontology.org) is a database that defines and describes the functions of genes and proteins. The GO database has three categories in total. Biological process (BP), cellular component (CC), and molecular function (MF) are the terms used to define the molecular tasks that gene products can perform as well as the cellular environment in which they live. The Kyoto Encyclopedia of Genes and Genomes (KEGG) (https://www.kegg.jp) is a database that may be employed to anticipate protein interaction networks for physiological activities as well as to comprehend the roles and routes of genetic variants. The functionality and route of 10 genes having the strongest association with STMN1 were investigated using the GO and KEGG databases.

## Data Availability

All data generated or analyzed during this study are included in this published article.

## Abbreviations

STMN1: Stathmin 1
HHC: Hepatocellular carcinoma
M6A: N6-methyladenosine
CpG: Cytosine guanine
HSC: Hepatic stellate cells
HGF: Hepatocyte growth factor
KPNA2: Karyopherin α2
THR: Thyroid hormone receptor
E2F1: E2F transcription factor 1
MC: Multicenter
EMT: Epithelial-mesenchymal transition
BLCA: Bladder urothelial carcinoma
BRCA: Breast invasive carcinoma
CESC: Cervical squamous cell carcinoma and endocervical adenocarcinoma
CHOL: Cholangiocarcinoma
COAD: Colonic adenocarcinoma
ESCA: Esophageal carcinoma
GBM: Glioblastoma multiforme
HNSC: Head and neck squamous cell carcinoma
KICH: Kidney chromophobe
KIRC: Kidney renal clear cell carcinoma
LUAD: Lung adenocarcinoma
PRAD: Prostate adenocarcinoma
LUSC: Lung squamous cell carcinoma
STAD: Stomach adenocarcinoma
THCA: Thyroid carcinoma
UCEC: Uterine corpus endometrial carcinoma
GEO: Gene Expression Omnibus database
TCGA: The Cancer Genome Atlas
GEPIA2: Gene expression profile interaction analysis
AFP: Alpha-fetoprotein
OS: Overall survival
RFS: Disease-free survival
DSS: Disease-specific survival
PFS: Progression-free survival
ROC: Receiver operating characteristic curve
AUC: Area under the curve
MHC: Major histocompatibility complex
PPI: Protein-protein interaction
GO: Gene Ontology
KEGG: Kyoto Encyclopedia of Genes and Genomes
FoxM1: Forkhead box M1
YAP1: Yes-associated protein 1
TFDP1: Transcription factor Dp-1
Akt: Protein kinase B
HPA: Human Protein Atlas
